# A Large Multifocal Aggressive Osteoblastoma of Mandible: an Immunohistochemistry Case Study Report

**DOI:** 10.5681/joddd.2014.009

**Published:** 2014-03-05

**Authors:** Vinuth D.P., Poonam Agarwal, Dilip Gadewar, Gunjan Dube, Rajesh Dhirawni

**Affiliations:** ^1^Assistant Professor, Department of Oral Pathology and Microbiology, Hitkarini Dental College and Hospital, Jabalpur, India; ^2^Assistant Professor, Department of Oral Medicine and Radiodiagnosis, Hitkarini Dental College and Hospital, Jabalpur, India; ^3^Professor and HOD, Department of Oral Pathology and Microbiology, Hitkarini Dental College and Hospital, Jabalpur, India; ^4^Reader, Department Oral Surgery, Hitkarini Dental College and Hospital, Jabalpur, India; ^5^Professor, Department Of Oral Surgery, Hitkarini Dental College and Hospital, Jabalpur, India

**Keywords:** Aggressive osteoblastoma, cytokeratin, immunohistochemistry, mandible, p53

## Abstract

Aggressive osteoblastoma (AO) is a benign osteoblastic tumor which is rare in the head and neck region. Clinical and histo-logical features are therefore overlap with other benign and low-grade malignant tumors. The aim of this article is to report and discuss the differential diagnosis of an aggressive osteoblastoma in the mandible. A 25-year-old male patient reported with pain and asymmetry on the left side of the face since 8 months previously. Radiographic evaluation showed a mixed lesion extending from approximately the lower left premolar to the third molar region. After incisional biopsy, resection with continuity defect was carried out. Microscopic findings showed woven bone and bony trabeculae with varied degrees of mineralization along with sheets of osteoblast cells. Immunohistochemistry showed that p53 and cytokeratin (CK) were negative and ki-67 index was 7%. Postoperative follow-up for 15 months showed no evidence of recurrence.

## Introduction

Osteoblastoma (OB) is a rare benign primary bone tumor, most often occurring in the posterior arch of vertebrae and long bones of the lower limbs, metacarpals, metatarsals and facial bones, including the jaw.^1^ In 1972 Dorfman examined 23 cases of osteoblastoma and reported four cases exhibiting recurrent behavior and histologic features that would advocate the name ‘aggressive osteoblastoma’ (AO).^2^

Conventional osteoblastoma (CO) commonly affects males in the second decade of life.^1^ AO of the head and neck region is rare. Due to its relative rarity, limited data is available on its incidence and distribution; however, AO affects older age group than CO, usually arising in the third or fourth decade. The radiographic appearance is similar to CO and a round to oval radiolucent area is infrequently demarcated by a sclerotic margin. Foci of patchy, cloud-like radiopacities may be seen within the lesion. More aggressive appearance including significant cortical expansion and destruction can be seen. AO lesions are clinically and radiographically larger (>4 cm) than lesions of CO (<4 cm).^1,3^

We report a diagnostically challenging case of multifocal aggressive osteoblastoma of the mandible with clinical, radiologic, histopathological and immunohistochemical findings. The clinico-histopathological differential diagnosis and immunohistochemical features potentially useful for refining this tumor are discussed.

## Case report

**Figure 1.**
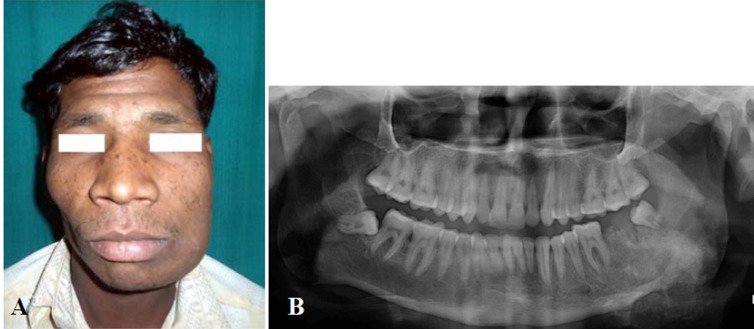
(A) Extra-oral photograph showing facial asymmetry on left side. (B) Pre-operative panoramic view show-ing ill-defined radiolucency on the left side with internal radiodensities extending approximately from 34 to 38 re-gion.

A 25-year-old male patient reported to the outpatient department with pain and asymmetry on the left side of the face since 8 months previously. History revealed that the swelling was initially small and progressively increased to the present size. The pain was a constant dull ache that exacerbated with function. Medical history was noncontributory. On clinical examination the swelling was firm involving the lower half of the left side of the face (Figure 1A). It was relatively well demarcated, tender and measured approximately 5×4 cm. The overlying skin was normal in color and texture, with no evidence of paresthesia. The submandibular and cervical lymph nodes were not palpable. Intraorally, a firm swelling was palpable in the buccal vestibule, extending from the lower left premolar (34) to retromolar region.

Panoramic radiographic examination revealed an ill-defined radiolucency with internal radiodensities extending from 34 to 38 regions (Figure 1B). Incisional biopsy was not conclusive. A tentative diagnosis of benign osteoblastic tumor was made. Under general anesthesia, an apron flap without lip split was used. Both the cortical plates of the mandible were infiltrated with tumor. Resection with continuity defect was carried out with distal bone cut just mesial to the canine and proximal bone cut from the sigmoid notch posteriorly to the posterior border of the mandible. Reconstruction was carried out using a 2.7-mm plate to stabilize the remaining portion of the mandible. The postoperative recovery was uneventful. The patient is under periodic follow-up; there is no evidence of recurrence after 15 months.

**Figure 2.**
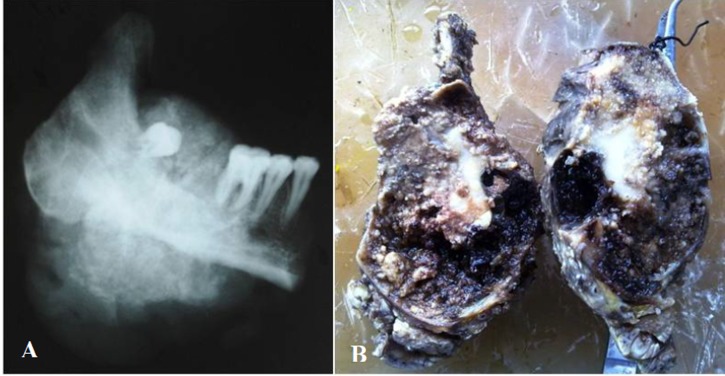
(A) Gross specimen showing central areas of tumor mass admixed with foci of vascularized regions (dark areas). (B) Specimen radiograph showing a mixed appearance.

**Figure 3.**
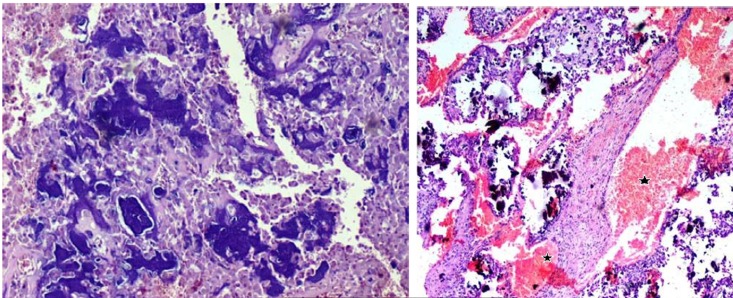
A: Trabeculae of immature woven bone (blue color) along with multiple layers of large epithelioid os-teoblasts (H&E; ×100). B: Intertrabecular spaces showing cellular and well vascularized spaces (^*^), which are rare in AO (H&E; ×100).

**Figure 4.**
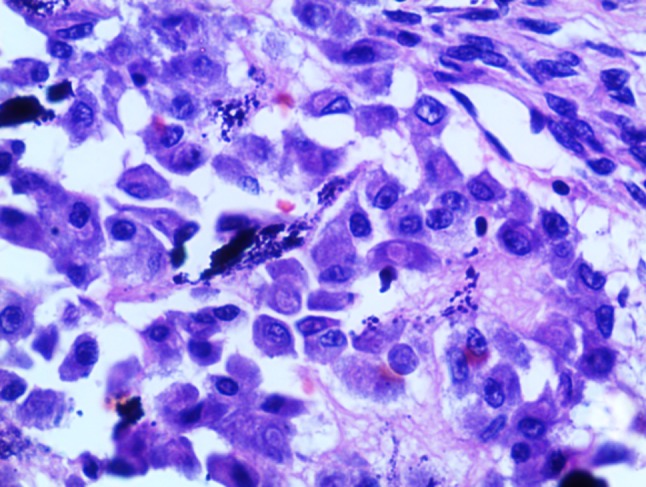
A sheet of osteoblasts with eccentric nuclei and abundant cytoplasm (H&E; ×400).

The excised specimen showed a relatively compact mass of reddish to brown friable tumor. Interestingly, dark brown hemorrhagic cyst-like areas were also noted (Figure 2A). The specimen radiograph showed diffuse areas of radiopacities (Figure 2B). Microscopically, cellular areas of large osteoblast cells, irregular shape of woven osteoid and trabeculae of varied thickness and degree of mineralization were seen (Figure 3A). Stroma that filled the intertrabecular spaces was well vascularized, resembling large cavernous spaces (Figure 3B) and few multinucleated giant cells were noted. Osteoblasts showed eccentric oval nuclei, prominent nucleoli and eosinphillic cytoplasm (Figure 4), with very few mitotic figures. Cellular atypia was absent. Multifocality (overall single lesion but with more than one nidus within a single bone) was apparent but permeation of host bone was not evident.

**Figure 5.**
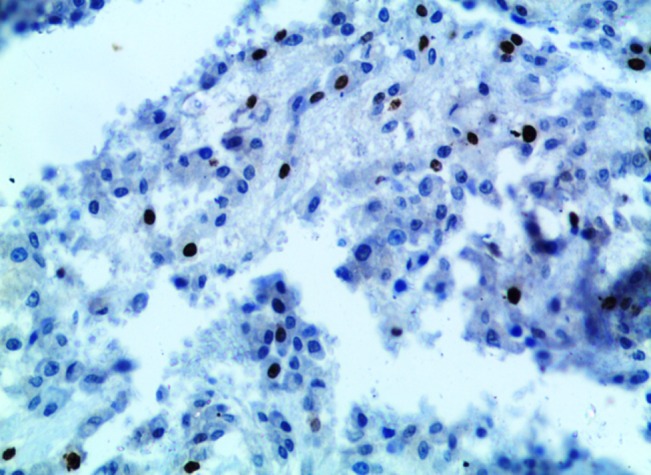
Immunohistochemical staining for Ki 67 showing diffuse and intense positivity of tumor cells (×100).

A series of immunohistochemical analyses was performed using monoclonal antibodies to Ki-67 antigen, p53 protein and cytokeratin (CK). Ki-67 labeling index was 7% (Figure 5), calculated after analyzing 1000 cells in 5 high-power fields in the region of the tumor with the greatest density of staining while p53 and CK were negative.

Based on these features, a final diagnosis of multifocal aggressive osteoblastoma was made.

**Figure 6.**
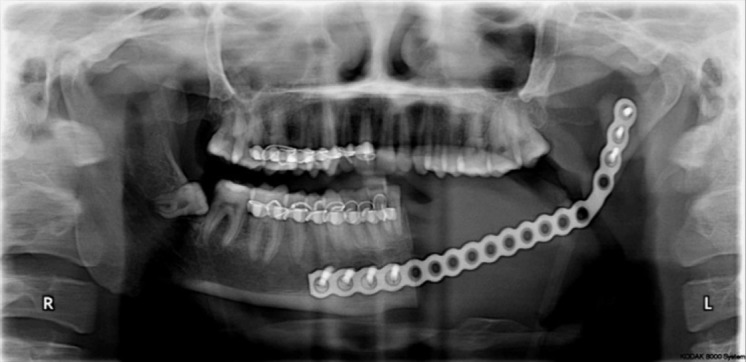
Post-operative panoramic view.

Postoperative follow-up for 15 months showed no evidence of recurrence (Figure 6).

## Discussion

Due to its rarity and close resemblance to other bony lesions of the jaws, AO presents a diagnostic challenge. The clinical-radiographic appearances are not diagnostic and may mimic other benign and malignant conditions. Histopathological differential diagnosis in such cases includes CO, low-grade osteosarcoma and cementoblastoma. Fibroosseous lesions and tumors of bony origin should also be considered for the same.

CO is biologically benign with limited growth potential. Histopathologically, it consists of delicate and lace-like trabeculae of osteoids lined by osteoblasts. The inter-trabecular stromal spaces show spindle-shaped fibroblasts, well-vascularized areas and multinucleated osteoclast-like giant cells. There is absence of mitotic figures and cellular atypia. Osteoblasts have moderate amounts of eosinophilic cytoplasm and a prominent round to oval nucleus. The soft tissue and bony component are usually at least in equal volume. The periphery of the lesion exhibits gradual merging of osteoid trabeculae with adjacent normal bone.^1,3^

Low-grade osteosarcoma (LGO) warrants separate recognition because its prognosis, unlike that of osteoblastoma, is excellent. It is a rare variant of osteosarcoma, accounting for 1.1-1.4% of all osteosarcomas. Clinical signs and symptoms are common between AO and LGO.^4^The radiographic features vary from lytic to sclerotic. Lesion borders can either be well-defined or ill-defined with cortical destruction. Histopathologically, spindle cell proliferation or large osteoblasts with hyperchromatic round to oval nuclei, prominent nucleoli and deeply-stained cytoplasm is seen. The cellular atypia is minimal, few abnormal mitoses ranging from 4 per 10 high-power fields. Bone production is irregular and scattered seams of osteoids embedded in a collagen stroma with multiple vascular spaces are seen. Most importantly tumor border shows infiltration and entrapment of host bone.^5^

In the head and neck region it is very difficult to make a diagnosis of osteoid osteoma based on clinical and radiographic features. Radiographically, lesions under 2 cm are osteoid osteomas, while osteoblastomas are larger. Microscopically, central distinct compact osteoid tissue nests are seen with varying degrees of calcification and constant perifocal osseous reaction. The nidus consists of richly-innervated fibrous stroma containing interconnected trabeculae of osteoid and woven bone lined with osteoblasts and osteoclasts. Ample vascular spaces and giant cells are frequently seen in osteoblastomas, while they are rare in osteoid osteomas.^6^

In 1985 Waldron suggested that another lesion which could easily be confused with osteoblastoma is cementoblastoma. Clinical and radiographic findings are very vital in such circumstances. Like cementoblastomas, osteoblastomas grow in close relationship with the adjacent tooth but cementoblastoma is fused to the roots and radiographic view shows a radiopaque mass merged with roots, obliterating the root outline as well as the periodontal ligament space. Histopathologically, there are interlacing trabeculae of mineralized tissue that resemble cellular cementum and have a more eosinophilic appearance compared to the characteristic basophilic trabeculae seen in osteoblastoma. Sometimes the cells are plump, angular and somewhat plasmacytoid, similar to osteoblastoma.^7^

Central ossifying fibroma is similar clinically and radiographically to osteoblastoma. Both can form immature bone, but the former is a painless, relatively uniform cellular proliferation of spindle-shaped fibroblastic cells which are more fibrous and less vascular and lack the presence of a large number of plump, actively proliferating osteoblasts cells.^3^

With regard to fibrous dysplasia, abnormal pigmentation of skin and endocrinopathies with altered serum alkaline phosphatase is usually present. Radiographic picture ranges from multilocular radiolucencies in the early stages to ground-glass/mixed appearance in mature stages.^6^The histological aspects are distinctive and characterized by irregularly shaped trabeculae of immature woven bone in a cellular matrix with loosely arranged fibrous stroma. The bone trabeculae are not connected to one another and are curvilinear in shape, leaving no room for doubt.^3,8^

In our case, there was no cellular atypia and permeative pattern; however, to predict the behavior and identify other morphological parameters immunohistochemical analysis was performed. Ki67 proliferative index was 7%, which was higher than previous reports.^8,9^ Bonar et al^10^ reported MIB (monoclonal antibody that identified the ki67 antigen) index up to 15%; a higher rate of MIB-1 supports the diagnosis of osteoblastoma-like osteosarcoma or LGO.^11^ CK and p53 immunostaining was negative. Previous results on p53 and osteoblastoma have shown great variations.^8,9,12^Oliveira et al^13^ investigated the biological behavior of classical and atypical osteoblastomas in comparison to osteosarcomas using PCNA, p53 immunohistochemistry and p53 gene mutations in classical, atypical osteoblastomas in comparison to osteosarcomas. Atypical osteoblastomas, osteosarcomas and tumor recurrence were statistically correlated with a high PCNA labeling index and p53 immunoexpression.

Although our case histologically showed epithelioid-shaped osteoblast cells, CK was negative as reported by Lennart Angervall and Román Carlos et al.^9,14^Epithelioid cell is a morphological term, and the cells do not necessarily express CK.^14^Since origin and differentiation of osteoblasts differs from epitheloid cells, we believe the term *“Epitheloid”* has been used erroneously in the literature. Pubmed search revealed no reports of aggressive osteoblastoma with CK immunohistochemical evaluation. To the best of our knowledge this is the first report of aggressive osteoblastoma with CK.

Conversely, in the present case we noted remarkable vascularized areas, which resembled telangiectatic spaces as reported by Lennart Angervall et al.^9^Treatment varies from curettage to en block resection. It depends on tumor size, site, extent of radiographic involvement and biological behavior. There are no evident histological features that provide clues to the biological behavior. Recurrence rate of CO is 13.6% and that of AO is 50%. En block resection is the treatment of choice in cases of AO.^2,8^

## Conclusion

AO shows a wide array of appearances microscopically; diagnosis can be a real challenge, especially with incisional biopsy. Reviews pertaining to nomenclature of osteoblastoma have been divisive due to their overlapping features and the relative rarity of the lesions. Terms like atypical osteoblastoma, epitheloid osteoblastoma, pseudo-malignant osteoblastoma and malignant osteoblastoma perplex the pathologist. The aggressive behavior should be predicted considering the size and radiological and histopathological features. Further studies are essential in IHC and molecular areas to understand the biological behavior of aggressive osteoblastoma.
